# Clinical Cases in Medical Wards of Mercy Hospital

**Published:** 1871-05

**Authors:** 


### Clinical Reports.

### CLINICAL CASES IN MEDICAL WARDS OF MERCY HOSPITAL.

Clinic by PROF. DAVIS. From Notes by S.

CASE I.—DISEASE OF KNEE-JOINT WITH LATENT TUBERCULOSIS.—This young man has been affected with ill-health for two or three years, becoming very much emaciated, blood impoverished, etc., with characteristic signs of inflammation at the knee-joint. Was brought to me some time since, with a special request that I should take charge of him; at that time had been for three or four months in the care of a surgeon in this city, who had decided that an amputation was necessary. The attention of all parties seemed to have been absorbed in the leg, as though the disease of the knee embodied all that was the matter.

I found a moderate degree of tumefaction about the knee, chiefly, I think, from a thickening of the synovial membrane, and a moderate degree of effusion, sufficient to give rise to slight fluctuation at the sides and above the patella, but no evidence of a carious condition of the bone. The patient is able to have the joint moved freely forwards and backwards—can bear considerable weight upon it, and walks with facility; but there are other reasons why I did not encourage him to go on and have the limb amputated. The knee-joint did not present evidence of actual disease sufficient to give rise to the general emaciation.

The patient is very anæmic—lips pale—pulse soft and compressible, with about the degree of emaciation which usually accompanies the second stage of tuberculosis, and has a slight cough—also a fair appetite, though losing flesh all the time—taking enough nourishment, if it was assimilated, to gain flesh every day and receive a good degree of strength, but only becoming more impoverished.

You may lay it down as an axiom, that will have few exceptions, that if you find a patient, notwithstanding absence of fever or acute local affection, with reasonable appetite, yet losing flesh, the blood growing thin and impoverished, that there is some change in the system which is of a serious organic character, and it will generally be found that the patient is laboring under a slow organic disease.

On examining the lungs I find a perceptible difference in the resonance of the two sides, and on applying the stethoscope observe a little irregularity in the progress of the inspiratory sound, and a renewal of it in the expiratory act—positive well-marked increased vibration of the voice, and a little atrophy of the left side of the chest. In the prolonged and slightly irregular respiratory murmur we find the earliest apparent physical signs of tubercular deposit in the lungs. A thing which points to disease of the mesentery glands, is the fact that the patient is taking a fair amount of food and not being nourished by it. He has also had, at frequent intervals, pains in the abdomen, a little to the right side, and while the limbs are attenuated, the abdomen is full—slightly tympanitic, and has a doughy feel.

During the last week or ten days there has been considerable tendency to diarrhœa, so that it has been necessary to use medicine directly for the purpose of counteracting it.

The patient complained of his throat being somewhat sore, but this did not attract much attention at first, and he was directed to use a simple wash—when, however, this did not relieve it, I was led to examine into the condition of the parts more particularly, and found a dark ulcerated patch of a purplish hue behind the parts, which, with the general depraved state of the blood, strongly inclined me to think that there was a hereditary syphilitic taint in addition to the tubercular deposit.

Directed a teaspoonful of the following mixture to be taken once a day:

R. Iodide Soda,------------------------ʒiij.

Bi Chld. Hydrag.,--------------------j gr.

Fl. Ext. Conium,---------------------℥j.

Syrup Orange Peel, ------------------℥iij. M.

The throat improved very rapidly, and in the course of two weeks was considered entirely well.

Complained of some pain in the knee-joint, which was entirely relieved by the application of a blister, which also reduced the swelling. The abdomen now seems a little full, and slightly tympanitic and tender—sound on percussion indicates clearly that it is not water or any solid tumor, but a state of fullness dependent upon imperfect assimilation. The sounds on auscultation indicate clearly the existence of latent tubercular deposits in the lungs.

If no congestion be determined in the parts, the patient may go on for years, and he be unconscious of the existence of any trouble about the lungs. But if he should take cold, or anything wake up a determination of the blood, it would be likely to establish a low grade of inflammatory action around this deposit, which, if not relieved in three or four weeks, would induce softening of the deposits, and the patient would begin to expectorate masses of yellow opaque matter, with mucus, etc.

In the treatment of this case we would rely on good air, plain food, the iodine alteratives, and such anodynes as would restrain irritation in the alimentary canal. Would not advise the long continued use of mercurial alteratives, but think the use of the alterative mixture containing the iodide of sodium and bi-chloride of mercury for a week at a time, perhaps once a month, would be useful. Up to the present time his bowels have been easily controlled by the turpentine and laudanum emulsion; and he has taken six grains of iodide of potassa morning and noon.

CASE II.—HEMIPLEGIA.—This patient had been in good health and was attacked suddenly with violent and well-defined pain in one side of the head, followed by fever and continued pain, which caused it to be styled brain fever, and lasted for a week or more, at the end of which time she was found to have complete hemiplegia, and was brought to the hospital.

On admission the pain had not entirely gone; pulse was slightly accelerated, not full, but strong; heat of the surface slightly elevated, with somewhat increased sensibility in the paralyzed parts.

Paralysis was observed over one entire side of the body; one-half of the tongue being slightly affected; the arm and leg, on that side, utterly destitute of motion, but control of the sphincters was still perfect.

It is the left side which is paralyzed, the pain in the head having been in the right side, at the junction of the frontal and right parietal bones.

The suddenness of the attack, the fever, and continued pain in the head, with hyperæsthesia and loss of motion, led me to attribute it to capillary congestion of the interior portion of the right anterior lobe of the brain, coming suddenly, developing severe pain, and leaving some degree of exudation; but probably not sufficient actual extravasation of blood to form a clot.

*Treatment.*—Either one prompt bleeding, or leeching, at the outset, before sufficient exudation had taken place to cause paralysis, would have exerted a good influence, especially if followed by sedatives to take off the increased force of the circulation, keeping the bowels open, cold to the head, etc., but the time for these remedies had passed by, and the patient is already paralyzed. She was accordingly put upon the following course of treatment:

℞. Potass. Bromide,......................ʒiv.

" Iodide,......................ʒij.

Water,......................℥iv. M.

One teaspoonful every four hours. Directed her to be kept at rest; the bowels to be moved with laxatives if necessary.

In the first few days there was no change, except a gradual decline of the pain in the head.

The next improvement observed was diminished hyperæsthesia, but without much indication of change in the motor power. In about ten days, however, she was able to stand, and from that began to walk, but in a very awkward manner, characteristic of this form of paralysis, carrying the foot forward by means of the muscles in the upper part of the thigh, the outer muscles seeming to be more paralyzed than those of the inner side of the limb, so that in walking the toes turn outwards.

After she began to walk, from some cause unknown, probably over-excitement of the mind, the pain came back in the head, which was subdued in two or three days by giving in addition to the other medicine, 20 grains bromide potassa with a little chloral, and directing her to be kept perfectly still.

Yesterday, for the first time, she was able to raise the arm.

The patient is a little too anxious, and will be apt to overdo, but if we can keep the nervous system composed, and induce regular and systematic exercise, she will doubtless gradually regain the use of the parts.

Our success in the management of these cases depends upon the proper adaptation of treatment to the successive stages of the disease.

In the use of the bromide and iodide we bring in an alterative, combined with a moderate sedative and quieting influence, allaying morbid sensibility, and promoting absorption; when this has been accomplished, we shall begin, very cautiously, little by little, with another class of remedies, *viz.*, nerve tonics, of which strychnine stands at the head.

The great thing is to know when to leave off the alterative and begin with the tonic treatment; if we commence the latter too quick, it will bring on a return of the pain in the head, prostration, fever, and loss of power; if deferred too long, the patient will cease to improve and become exhausted, grow pale and feeble.

Treatment should be so conducted as to remove the inflammatory action, and get rid of the exudation, then, and not before, commence giving the other class of remedies.

I have usually withheld tonics as long as hyperæsthsia or muscular rigidity remained.

You will notice in the early stage of paralysis, from inflammatory action especially, that hyperæsthesia will be the rule, with more or less actual stiffening or rigidity in the paralyzed part. As long as these conditions exist, you are safe in acting upon the rule, that no benefit will be derived from the use of strychnine, or the different varieties of electricity, galvanism, and other stimulating agents.

When hyperæsthesia ceases, and there is flaccidity of the muscles, you will find it advisable to gradually diminish the sedatives and alteratives, and commence the use of nerve tonics; feeling your way carefully by introducing the battery, very carefully at first, giving moderate doses of strychnine, $\frac{1}{60}$ grain, morning and night, while you continue the bromide and iodide after breakfast, and dinner, and at bedtime.

If this is well borne, the potassium mixture may be limited to supper and bedtime, giving the strychnine three times a day, afterwards increasing the dose to $\frac{1}{40}$ grain, and giving the solution of the bromide at bedtime only, which will contribute to sleep and keep the secretions free.

In this way a full restoration to power and normal sensibility may be brought about, usually in from one to three months.

We shall continue to pursue the present order of treatment in the manner alluded to.

The question as to the probability of a full and perfect recovery, depends on whether positive sanguinous extravasation has taken place; if not, the mere inflammatory exudation can be made to be reabsorbed and function restored; if sanguinous, we shall be unable to bring about resorption of the fibrinous material, the brain in the vicinity will remain hardened, and its functions permanently suspended, ultimately proving fatal from altered nutrition and disintegration of brain substance.

Case III.—Chronic Inflammation of the Stomach and Œsophagus.—This patient is a young German, whose business has been barkeeping. He has not drank what is called to excess, but daily taking more or less alcoholic drink.

The combined influence of this habitual use of liquor, and too much confinement, has engendered well-marked gastric irritation, probably genuine follicular inflammation, but of a very low grade, just sufficient to give rise to an increased secretion of gastric fluid, and vomiting, or what is called pyrosis, every morning before breakfast.

In addition there is, more or less distress after taking food, which produces a heavy sensation in the stomach, and is sometimes rejected.

Besides these symptoms there is a difficulty in swallowing, so that extra effort is required, and it hurts, as if there was a sore place in the œsophagus; there is a degree of actual inflammation of a chronic character in the lining membrane of the pharynx and œsophagus, similar to that existing in the follicles of the stomach.

I have noticed, occasionally, from the commencement, a peculiar noise in his breathing, a coarse rough sound, and he complains of not getting breath good, of a want of air. Three-fourths of the respirations seem natural in sound, but if he takes a quick breath it will bring a vibration in the vocal apparatus, giving a peculiar sound with the ingress of air, and this may sometimes be detected almost to the bottom of the chest. This led me to examine more closely to see if there was any evidence of a tumor or enlargement of the aorta which would exert mechanical pressure upon the trachæ, but I was unable to discover anything of the kind. The chest expands easily.

I think there is a degree of direct nervous irritation that is reflected to the larynx, causing tension of the vocal apparatus, and a sense of constriction that extends more or less to the tubes below.

The tongue is red along the edges and at the tip, the papilla a little prominent, but not much coated; lips red; face flushed, and covered with an eruption resembling psoriasis, a form of tetter, consisting in slight tumefaction of the skin, with desquamation of the cuticle; this appeared within the last few days.

The membrane covering the tonsils and lining the pharynx is a little reddened, and the parts are swollen and tumefied.

When admitted, complaining of these symptoms, and learning his previous history, I directed a powder of

Bismuth Subnit.,--------------------grs. vj.
Lupuline,----------------------------grs. ij.

To be taken three times a day, before meals, and to hasten the disappearance of the inflammation in the stomach, I prescribed, in addition, the following solution:

℞. Carbolic Acid,----------------------grs. vj.
Water,--------------------------------℥ij.
Tinct. Opii et Camph.,--------------℥jss.
Glycerine,--------------------------℥ss. M.

One teaspoonful to be given after each meal.

The patient improved to a certain extent, vomiting less frequently, but the difficulty of swallowing, difficult breathing, etc., remained; treatment so far had not seemed to make much impression on that part of the difficulty.

Two days ago, when this eruption on the face made its appearance, and the membrane of the pharynx assumed a darkish hue, I was led to believe that there might be another element present, which had operated to produce a degree of impurity of the blood, of a specific character.

I accordingly directed, in place of the carbolic acid solution, the following:

℞. Iodide Sodium,------------------------ʒiij.

Hydrag. Bichlo.,------------------------gr. j.

Fl. Ext. Conium,------------------------℥j.

Syr. Licorice,--------------------------℥iij. M.

One teaspoonful after each meal, the powders of bismuth, etc., to be continued as before.

He has not been on this long enough to develop decided effects from it, but if it does not set up an irritation of the stomach, it will not be a week before this eruption and much of the bad feeling in the upper part of the pharynx and œsophagus will disappear, by that time the powders should be limited to twice a day, at breakfast and dinner, giving the liquid mixture as before; meanwhile the diet should be carefully guarded.

